# Winner’s curse in rare variant analysis: effect size estimation bias depends on effect direction and the association method used

**DOI:** 10.3389/fgene.2025.1416673

**Published:** 2025-08-08

**Authors:** David Soave, Melisa Hayalioglu, Lei Sun

**Affiliations:** ^1^ Department of Mathematics, Wilfrid Laurier University, Waterloo, ON, Canada; ^2^ Ontario Institute for Cancer Research, Toronto, ON, Canada; ^3^ Department of Statistical Sciences, University of Toronto, Toronto, ON, Canada; ^4^ Division of Biostatistics, Dalla Lana School of Public Health, University of Toronto, Toronto, ON, Canada

**Keywords:** genome-wide association study, rare variants, joint analysis, estimation, selection bias, winner’s curse, effect heterogeneity

## Abstract

For complex human traits, a large portion of genetic heritability remains unaccounted for beyond common genetic variants; therefore, estimating the contribution of rare variants (RVs) to the etiology of complex traits is of interest. Research in this domain has primarily focused on gene-based RV testing methods, in which information from multiple variants is combined to maximize statistical power in detecting genes associated with the trait of interest. However, after discovering an association, estimating individual effects becomes challenging due to sample size limitations. Hence, the focus may shift to estimating the average genetic effect (AGE) for the group of RVs analyzed. This study demonstrates that both AGEs and individual variant effects can be influenced by competing *upward* and *downward* biases, resulting from the winner’s curse and the heterogeneity of individual variant effects, respectively. Various bias-correction techniques, including bootstrap resampling and likelihood-based methods, have been proposed to address the winner’s curse bias. We conduct a simulation study to illustrate the ramifications of these competing biases on variant effect size estimation and how they complicate the precision of pooled estimates obtained from different bias-correction techniques. We then examine the individual effect estimates of the causal variants across the simulation replicates to show how they may contribute to the observed upward and downward biases when RVs are pooled.

## 1 Introduction

Common genetic variants generally account for only a portion of genetic heritability (Pritchard, 2001; Bodmer and Bonilla, 2008; Visscher et al., 2017). One of the proposed explanations for this missing heritability is that genetic architectures are highly polygenic, involving numerous variants with individually small effects that collectively contribute to complex traits (Park et al., 2010). Park et al. (2010) demonstrated that the distribution of effect sizes in genome-wide association studies (GWASs) follows an exponential-like decay, suggesting that many susceptibility loci remain undetected due to limited power in current study designs. Furthermore, linkage disequilibrium (LD) structure plays a key role in the proportion of heritability captured by GWASs as causal variants that are poorly tagged by genotyped markers lead to the underestimation of genetic contributions (Yang et al., 2012). These challenges are particularly relevant for rare variants (RVs), which, despite potentially larger effect sizes, remain difficult to detect due to their low minor allele frequency (MAF) and weaker LD with neighboring markers (Manolio et al., 2009; Wang et al., 2021).

To improve detection power, gene-based or pooled association tests have been developed to aggregate information across multiple RVs, allowing for the joint evaluation of their contribution to trait variation (Morgenthaler and Thilly, 2007; Li and Leal, 2008; Madsen and Browning, 2009; Price et al., 2010; Neale et al., 2011; Wu et al., 2011). Most of these gene-based RV tests belong to either the class of linear tests (e.g., the burden test) or the class of quadratic tests (e.g., variance components or SKAT methods) (Derkach et al., 2014). As each type of test can be more powerful under different scenarios, hybrid tests have also been proposed to combine the association evidence from two or more complementary approaches (Lee et al., 2012; Derkach et al., 2013; Liu et al., 2019).

Following association testing, it is of interest to estimate and understand the genetic effect sizes of the significant RVs. The gene-based approach, although effective at association testing, limits our ability to estimate the *individual* genetic effect size (β) for each RV. A common workaround approach to effect size estimation in this setting is to consider the average genetic effect (AGE) and estimate $\beta_{\text{AGE}}$ , the effect of one single collapsed genotype variable (Liu and Leal, 2012). However, this approach has limitations as the group of RVs analyzed likely contains some null variants not associated with the trait of interest. Additionally, among the truly associated RVs, their effect directions may differ. Subsequently, the inclusion of null RVs or RVs with effects in opposite directions may lead to a *downward* bias of the effect size estimate; this downward bias is underappreciated in the literature. On the other hand, hypothesis testing and effect estimation performed using the same sample lead to an *upward* bias due to the well-known phenomenon of the winner’s curse or selective inference (Göring et al., 2001; Sun and Bull, 2005; Efron, 2011; Taylor and Tibshirani, 2015).

For the analysis of individual common variants, several approaches have been proposed to correct for the winner’s curse, including bootstrap resampling (Sun and Bull, 2005; Faye et al., 2011), likelihood-based approaches (Zöllner and Pritchard, 2007; Ghosh et al., 2008; Zhong and Prentice, 2008; Xiao and Boehnke, 2011), and Bayesian approaches (Xu et al., 2011). More recently, additional work has explored the winner’s curse and proposed methods for correcting estimation in genome-wide association and polygenic risk score studies, along with eQTL and mediation analyses (Shi et al., 2016; Huang et al., 2018; Hu et al., 2019; Xie et al., 2021; Yang et al., 2021).

Focusing on the joint analysis of multiple rare variants using the combined multivariate and collapsing (CMC) method of Li and Leal (2008), Liu and Leal (2012) estimated $\beta_{\text{AGE}}$ using a modified bootstrap method, where the median of all bootstrap-based estimates was used instead of the mean, as employed by Sun and Bull (2005). Although they showed that the proposed method provides reasonable bias-reduced $\beta_{\text{AGE}}$ estimates, the following questions require further investigations: (1) Do the original bootstrap and likelihood methods perform equally well under the simulation scenarios considered? (2) Do the individual RV effect estimates suffer equally from the winner’s curse, regardless of their true effect sizes or effect directions? (3) Will the biases depend on the type of test used (e.g., linear vs. quadratic)? This report aims to answer these questions through a simulation study.

Our investigation builds upon the recent work by Grinde et al. (2017), with some notable distinctions. First, while Grinde et al. (2017) focused on case–control studies and used the squared difference of MAFs between cases and controls as the measure for an individual variant effect, we use a conventional effect size measure, which is the rate of change in outcome (either binary or continuous) per unit change in the genotype. Second, we consider both the individual variant effects and the average genetic effect. Finally, while Grinde et al. (2017) centered on the overestimation problem due to the winner’s curse, our study further considers the competing downward bias due to effect heterogeneity, providing a more nuanced understanding of the biases inherent in pooled analysis of multiple rare variants.

The remainder of this paper is organized as follows. Section 2 reviews existing methods, including association testing of rare variants, parameter estimation of the average genetic effect $\beta_{\text{AGE}}$ , and the winner’s curse. Section 3 presents a simulation study examining the complex biases in effect size estimation for rare variants, including the previously underappreciated ramification of effect heterogeneity between variants in this study setting. Section 4 provides some concluding remarks.

## 2 Methods

### 2.1 Brief review of joint association testing of multiple rare variants

As association analyses between individual rare variants (X) and a complex trait (Y) have low power, gene-based pooled tests have been proposed. These tests aggregate the trait–genotype association information across multiple RVs and jointly test the null hypothesis that the trait is independent of the group of RVs. Let $Y_{i}$ be the trait value across n subjects, where i=1,…,n , and $X_{ij}$ be the genotypes for a group of J rare variants, where j=1,…,J , and $X_{ij}$ is generally 0 or 1, representing the absence or presence of the rare minor allele. Consider the set of association scores for the J RVs,

$$S_{j}=\underset{i}{\sum }\left(Y_{i}-\bar{Y}\right)X_{ij},\;j=1,\ldots ,J.$$

There exist two classes of score tests that aggregate these scores in different ways (Derkach et al., 2013). The linear class $T_{L}$ obtains the weighted average of $S_{j}$ ,

$$T_{L}=w^{T}S={\left(w_{1},\ldots ,w_{J}\right)}^{T}S=\overset{J}{\underset{j=1}{\sum }}w_{j}S_{j}.$$

Depending on how the weights $w_{j}^{\prime }s$ are defined for the individual variants, methods in this linear class include the CAST (Morgenthaler and Thilly, 2007), the weighted sum statistic (WSS) (Madsen and Browning, 2009), and the variable threshold (VT) methods (Price et al., 2010). These methods are most powerful when all or almost all aggregated RVs are causal and have the same direction of effect, but they are sensitive to the signs of $S_{j}$ .

In contrast, the quadratic class $T_{Q}$ , in essence, obtains the weighted average of $S_{j}^{2}$ . More generally, let A be a positive definite (or semi-definite) symmetric matrix, then

$$T_{Q}=S^{T}AS,$$

where if A is a main-diagonal matrix $\left\{w_{j}\right\}$ , it is easy to see that $W_{Q}=\sum_{j=1}^{J}w_{j}S_{j}^{2}$ . Methods in this quadratic class include the C-Alpha (Neale et al., 2011), SKAT (Wu et al., 2011), and Hotelling’s T test. Based on the inherent squaring of the individual association scores, this class of tests is robust to bidirectional effects across the J variants but can lose power in the absence of effect heterogeneity.

Hence, there exists another class of methods that can integrate the linear and quadratic statistics, such as the SKAT-O method (Lee et al., 2012), Fisher’s method (Derkach et al., 2013), or the more recent ACAT approach (Liu et al., 2019). Penalized regression approaches, which allow grouping of multiple regions at once, form yet another class of methods.

For the purpose of this study, we examine the linear and quadratic classes to better characterize the key factors contributing to the estimation bias. Within the two classes, without loss of generality, we then focus on the equal weighting methods, namely, CAST (Morgenthaler and Thilly, 2007) and C-Alpha (Neale et al., 2011), from the linear and quadratic classes, respectively. Both are unweighted methods from their respective classes, providing a straightforward interpretation of results. To facilitate the study of $\beta_{\mathrm{AGE}}$ , the parameter of interest (Liu and Leal, 2012), we further consider the CMC RV testing method (Li and Leal, 2008), which we discuss in the following section.

### 2.2 Average genetic effect and CMC RV testing

It is usually desirable for investigators to estimate the genetic effect size after achieving a significant association testing result. This is commonly done for sample size estimation to conduct sufficiently powered replication studies. For the analysis of rare variants, Liu and Leal (2012) considered the following genetic model:

$$Y_{i}=\alpha +\underset{c\in C}{\sum }\beta_{c}X_{ic}+\varepsilon_{i},$$

where C is the set of truly associated RVs affecting the trait; the trait is assumed to be normally distributed for simplicity but without loss of generality. The parameters of interest for estimation are $\beta_{c}$ , along with the trait variance explained by the group of causal RVs, $\sigma_{G}^{2}=Var\left(\sum_{c\in C}\beta_{c}X_{ic}\right)$ .

Unfortunately, due to the same logic that limits power in detecting causal SNPs individually, we are unable to estimate the individual $\beta_{c}$ efficiently. Additionally, in practice, it is not possible to estimate these parameters directly as we are unable to distinguish between the causal and non-causal variants.

Possibly, the most organic choice of the parameter estimation method when association tests are based on weighting/collapsing variants is the AGE, defined as the change in the trait value per unit change in multivariate genotype coding $K\left(X_{i}\right)$ (Liu and Leal, 2012). Thus, the following fitted model (Equation 1) is used for the inference and estimation of $\beta_{\mathrm{AGE}}$ of a group of J rare variants:

$$Y_{i}=\alpha +\beta_{\mathrm{AGE}}K\left(X_{i}\right)+e_{i},$$ (1)

where $\beta_{\mathrm{AGE}}$ corresponds to the effect of a single collapsed genotype variable K .

Under the CMC RV association method (Li and Leal, 2008), testing the null hypothesis $H_{0}:\beta_{\mathrm{AGE}}=0$ involves using an indicator function $K\left(X_{i}\right)=I\left\{\left(\sum_{j\in J}X_{ij}\right)>0\right\}$ , which denotes the presence of the minor allele at any of the J rare variants analyzed jointly. As the set of J RVs included in the analysis and the set of C truly associated RVs may not be the same and $\beta_{j}=0$ for a null RV, Liu and Leal (2012) showed that under the CMC method,

$$\beta_{\mathrm{AGE}}=\frac{\sum_{j\in J\cap C}\beta_{j}q_{j}}{\sum_{j\in J}q_{j}},$$ (2)

where $q_{j}$ is the MAF of variant j . Finally, we note that, although the CMC RV testing method does not fit into the three classes (linear, quadratic, and hybrid) discussed in Section 2.1, it is fundamentally a linear type of test as it is sensitive to the direction of the genetic effect, as evident from Equation 2 and the results in Section 3.

### 2.3 Winner’s curse and bias-corrected estimates

When analyzing individual common variants that have been filtered and selected based on statistically significant association, it is well established that the effect size estimate, ${\hat{\beta }}_{\mathrm{naive}}$ , obtained from the same sample used for association testing, tends to overestimate the true effect size of the variant unless the association test has 100% power (Göring et al., 2001). This upward estimation bias is also known as the winner’s curse. To correct for the winner’s curse, several approaches have been established, including likelihood-based, resampling, and Bayesian, among which the bootstrap resampling approach is one of the most flexible approaches (Sun et al., 2011) and has been adapted for the rare variant setting (Liu and Leal, 2012).

The bootstrap approach, in essence, splits the sample into two independent sub-samples, using one for hypothesis testing and the other for parameter estimation (Sun and Bull, 2005; Faye et al., 2011; Sun et al., 2011). In brief, for each bootstrap sample, a complete GWAS is conducted to determine the associated variants of interest. For each associated variant identified in the bootstrap sample, its effect size β is estimated twice. First, using the same bootstrap sample, we obtain ${\hat{\beta }}_{D}$ , which mimics the original GWAS procedure for ${\hat{\beta }}_{\mathrm{naive}}$ . Second, using the remaining sample (consisting of individuals *not* selected for the bootstrap sample), we obtain ${\hat{\beta }}_{E}$ directly *without* requiring significant association results, thus avoiding the winner’s curse. The difference between the two estimates, ${\hat{\beta }}_{D}-{\hat{\beta }}_{E}$ , reflects the estimation bias, $\Delta_{\beta }$ , owing to the winner’s curse. To stabilize the sampling variation, Sun and Bull (2005) recommended repeated bootstrap sampling and taking the average, ${\hat{\Delta }}_{\beta }=\frac{1}{B}\sum_{b=1}^{B}\left({\hat{\beta }}_{D_{b}}-{\hat{\beta }}_{E_{b}}\right)$ . Finally, subtracting the average bias from the original-sample naive parameter estimate provides a bootstrap-corrected parameter estimate, ${\hat{\beta }}_{\mathrm{boot}}={\hat{\beta }}_{\mathrm{naive}}-{\hat{\Delta }}_{\beta }$ .

For rare variants, Liu and Leal (2012) investigated the flexible bootstrap correction approach (using the median, instead of the mean, of $\left\{{\hat{\beta }}_{D_{b}}-{\hat{\beta }}_{E_{b}}\right\}$ ) for $\beta_{\mathrm{AGE}}$ estimation under the CMC method. After first demonstrating the winner’s curse bias in unadjusted ${\hat{\beta }}_{\mathrm{AGE}}$ , Liu and Leal (2012) then showed that the bootstrapping method works well in correcting for the winner’s curse across several scenarios involving the pooling of causal and non-causal rare variants in the analysis.

The following simulation study extends the investigation of Liu and Leal (2012) in several ways. First, we examine whether the original bootstrap procedure of using the mean of $\left\{{\hat{\beta }}_{D_{b}}-{\hat{\beta }}_{E_{b}}\right\}$ works equally well for estimating the bias. Second, we compare the bootstrapping correction with the approximate likelihood-based approach $\left({\hat{\beta }}_{\mathrm{like}}\right)$ of Ghosh et al. (2008). Unlike the bootstrap method, which relies on resampling techniques to estimate bias, the Ghosh likelihood method corrects for bias using an approximate conditional likelihood. This method relies on the use of reported estimates of genetic effect and its standard error, facilitating correction of bias without requiring access to the original data. Finally, we investigate the role of effect heterogeneity across the different rare variants, including the previously underappreciated downward bias due to opposing effect directions. These are important considerations as researchers attempt to quantify the usefulness of bias adjustment procedures for parameters, such as $\beta_{\mathrm{AGE}}$ , for studies of rare variants.

To formalize the approach, we define the standardized effect size as follows:

$$\mu =\frac{\beta }{\hat{\text{SE}}\left(\hat{\beta }\right)}.$$

Given an observed estimate $\hat{\beta }$ and its standard error $\hat{\text{SE}}\left(\hat{\beta }\right)$ , the naive test statistic follows

$$Z=\hat{\mu }=\frac{\hat{\beta }}{\hat{\text{SE}}\left(\hat{\beta }\right)}∼N\left(\frac{\beta }{\hat{\text{SE}}\left(\hat{\beta }\right)},1\right).$$

Since only values where |Z|>c are observed due to selection, this induces bias in $\hat{\mu }$ . The Ghosh method corrects for this bias by modeling the *conditional likelihood*:

$$L_{c}\left(\mu \right)=p_{\mu }\left(z||Z|>c\right)=\frac{p_{\mu }\left(z\right)}{P_{\mu }\left(|Z|>c\right)}=\frac{f\left(Z-\mu \right)}{F\left(-c+\mu \right)+F\left(c-\mu \right)},$$

where f(⋅) and F(⋅) denote the *standard normal density* and *cumulative distribution functions*, respectively, (Ghosh et al., 2008). The bias-corrected effect size is then obtained by maximizing $L_{c}\left(\mu \right)$ , ensuring that estimation explicitly accounts for the selection process. This approach provides a correction that does not require access to individual-level genotype data. Instead, it relies only on summary statistics, such as reported effect sizes, standard errors, and significance thresholds, making it computationally efficient for large-scale GWASs and meta-analyses.

## 3 Simulation study

We conducted a simulation study using the Genetic Analysis Workshop 17 (GAW17) data (Almasy et al., 2011). The GAW17 “mini-exome” dataset consists of real human DNA sequence data from the 1000 Genomes Project (Consortium et al., 2010) and various qualitative and quantitative phenotype data simulated by the GAW17 data committee. For each phenotype, 200 replicate samples were generated by simulating different phenotype data based on the true genotype–phenotype model, conditional on the observed genotype data.

For this investigation, the sample for analysis consisted of the n=321 subset of unrelated Asian subjects (Han Chinese, Denver Chinese, and Japanese), and the quantitative trait Q2 was chosen for illustration without loss of generality. Among the 13 genes influencing the trait, method evaluation focused on the *SIRT1* gene, for which the best power possible is approximately 50% at the 0.05 level. This gene has a total of J=11 rare variants (MAF ≤ 1%), among which C=4 are assumed to be causal with effects in the same direction (i.e., the unidirectional scenario) in the GAW17 simulation, which we will extend to study bidirectional effects; parameter details are provided in Table 1. First, to improve inference stability, instead of analyzing the 200 replicates provided by GAW17, 2,000 new replicate samples were created based on the true SNP effect sizes from the four casual SNPs within *SIRT1* for Q2. Second, to examine the impact of effect heterogeneity, we allow one of the four variants to have an effect in the opposite direction (i.e., the bidirectional scenario).

**TABLE 1:** GAW17 simulation summary statistics for $\beta_{\mathrm{AGE}}$ AGE estimates. $\beta_{\mathrm{AGE}}$ is the true underlying AGE of all 11 SNPs under the CMC regression model 1 and calculated using Equation (2). ${\hat{\beta }}_{\mathrm{all}}$ reflects the estimate of $\beta_{\mathrm{AGE}}$ , with the sample average (SE) calculated over all 2,000 simulation replicates; ${\hat{\beta }}_{\mathrm{naive}}$ , ${\hat{\beta }}_{\mathrm{boot}}$ , and ${\hat{\beta }}_{\mathrm{like}}$ reflect the averages (SEs) over only significant replicates (where CMC Wald-test p<0.05 ), for the naive method of taking the same average of $\beta_{\mathrm{AGE}}$ directly and the bootstrap- and likelihood-based bias correction methods, respectively, as described in Section 2.3. The underlying individual effect $\beta_{j}$ (MAFs $q_{j}$ ) = [0(0.0016), 0(0.0047), 0.83(0.0031), 0.97(0.0016), 0(0.0016), 0(0.0016), 0(0.0016), 0(0.0016), 0(0.0031), 0.93(0.0016), 0.53(0.0047)], where, under the bidirectional scenario, the sign for $\beta_{10}$ is flipped. See Figure 1 for a visual display of the results.

Description	Four causal in the same direction	Four causal with 1° in the opposite direction
$\beta_{\text{AGE}}$	0.30	0.19
Estimation, mean (SE), before significance testing across all 2,000 replicates		
${\hat{\beta }}_{\text{all}}$	0.34 (0.27)	0.22 (0.27)
Estimation, mean (SE), after significance testing across 528 or 254 replicates		
	Power = 528/2000 = 26.4%	Power = 254/2000 = 12.7%
${\hat{\beta }}_{\text{naive}}$	0.67 (0.14)	0.64 (0.16)
${\hat{\beta }}_{\text{boot}}$	0.40 (0.24)	0.33 (0.24)
${\hat{\beta }}_{\text{like}}$	0.36 (0.25)	0.31 (0.23)

### 3.1 Setup

For each replicate sample, we conducted a linear regression analysis of the phenotype on the collapsed genotype parameter under the CMC pooling method (1), yielding an effect estimate $\left({\hat{\beta }}_{\mathrm{AGE}}\right)$ and corresponding Wald test p -value. To demonstrate the winner’s curse bias in the rare variant setting, we compared the distribution of the estimated ${\hat{\beta }}_{\mathrm{AGE}}$ from all 2000 replicates to that restricted to replicates with association p -values ≤0.05 . The choice of the liberal type-1 error level 0.05 was based on the overall low power of detecting these genes due to the small sample size (n=321) , small genetic effect (individual variant $\beta_{j}$ ranges from 0.53 to 0.97), very small MAF (ranges from 0.003 to 0.009), and the low proportion of the causal variants within the gene ( 4/11=36% causal); recall that bias increases as power decreases, so more stringent type-1 error levels would lead to greater biases.

Next, we assessed the performance of the bootstrapping approach for effect size adjustment proposed by Liu and Leal (2012) (described in Section 2.3) and the approximate likelihood-based adjustment method proposed by Ghosh et al. (2008) by applying them to each of the statistically significant simulation replicates identified.

To identify the contribution of each of the four causal RVs to the pooled genotype testing procedures and the $\beta_{\mathrm{AGE}}$ parameter estimates, the phenotype was regressed on each causal variant separately, yielding individual variant effect estimates $\left({\hat{\beta }}_{j},\;j=1,\ldots ,4\right)$ for each simulated replicate. We conducted this analysis based on the results (significance testing) of CMC (Li and Leal, 2008), CAST (Morgenthaler and Thilly, 2007), and C-Alpha (Neale et al., 2011) RV testing approaches. The CMC method was used by Liu and Leal (2012) for bias correction in the RV setting, and CAST reflects the linear class of combined test statistics, whereas C-Alpha falls within the quadratic class, providing an interesting comparison. It should also be pointed out that the CMC method can be viewed as a linear type of test; thus, its performance is expected to be characteristically similar to CAST.

We note that the effect sizes ( $\beta_{j}$ ) were assigned as part of the GAW17 simulation framework and do not necessarily reflect empirical estimates from GWASs or exome sequencing studies. Thus, in addition to the setup derived from GAW17, we extended our simulation study to explore additional scenarios with a higher proportion of causal variants. Specifically, 73% (8/11) of the variants were assigned causal effects, with individual $\beta_{j}$ values ranging from 0.53 to 0.97. Additionally, we considered cases where certain SNPs had substantially larger effect sizes, with 36% (4/11) of the variants exhibiting $\beta_{j}$ values between 0.53 and 3.0. These extensions aim to enhance the generalizability of our findings to a broader range of practical scenarios. In particular, rare causal variants, such as protein-truncating variants, are often associated with very large effect sizes (Decker et al., 2017).

### 3.2 Performance of the bootstrapping and likelihood effect size adjustments

Summary statistics for the estimate of $\beta_{\mathrm{AGE}}$ across the GAW17 simulation replicates are reported in Tables 1–3. We observed that the following results are qualitatively similar across the scenarios explored, and thus, we focus our discussion on Table 1. Consistent with the study by Derkach et al. (2013), the estimated power for this linear-type test is greater when causal variants all share the same direction of effect (power =0.26 ) compared to when variants with effects in the opposite direction are present (power =0.13 ). This corresponds to 528 statistically significant (p<0.05) replicates available for investigation under the unidirectional variant effects scenario and 254 under the bidirectional scenario (Table 1).

**TABLE 2:** GAW17 simulation summary statistics for $\beta_{\mathrm{AGE}}$ AGE estimates; very large effect sizes. $\beta_{\mathrm{AGE}}$ is the true underlying AGE of all 11 SNPs under the CMC regression model 1 and calculated using Equation (2). ${\hat{\beta }}_{\mathrm{all}}$ reflects the estimate of $\beta_{\mathrm{AGE}}$ , with the SE calculated over all 2,000 simulation replicates; ${\hat{\beta }}_{\mathrm{naive}}$ , ${\hat{\beta }}_{\mathrm{boot}}$ , and ${\hat{\beta }}_{\mathrm{like}}$ reflect the averages (SEs) over only significant replicates (where CMC Wald-test p<0.05 ) for the naive method of taking the same average of the $\beta_{\mathrm{AGE}}$ directly and the bootstrap- and likelihood-based bias correction methods, respectively, as described in Section 2.3. The underlying individual effect $\beta_{j}$ (MAFs $q_{j}$ ) = [0(0.0016), 0(0.0047), 0.83(0.0031), 2.0(0.0016), 0(0.0016), 0(0.0016), 0(0.0016), 0(0.0016), 0(0.0031), 3.0 (0.0016), 0.53(0.0047)], where, under the bidirectional scenario, the sign for $\beta_{10}$ is flipped.

Description	Four causal in the same direction	Four causal with 1° in the opposite direction
$\beta_{\text{AGE}}$	0.49	0.13
Estimation, mean (SE), before significance testing across all 2,000 replicates		
${\hat{\beta }}_{\text{all}}$	0.55 (0.27)	0.15 (0.27)
Estimation, mean (SE), after significance testing across 528 or 254 replicates		
	Power = 1,058/2000 = 52.9%	Power = 149/2000 = 7.5%
${\hat{\beta }}_{\text{naive}}$	0.75 (0.16)	0.57 (0.31)
${\hat{\beta }}_{\text{boot}}$	0.44 (0.30)	0.20 (0.23)
${\hat{\beta }}_{\text{like}}$	0.50 (0.30)	0.27 (0.23)

**TABLE 3:** GAW17 simulation summary statistics for $\beta_{\mathrm{AGE}}$ AGE estimates; many casual variants. $\beta_{\mathrm{AGE}}$ is the true underlying AGE of all 11 SNPs under the CMC regression model 1 and calculated using equation (2). ${\hat{\beta }}_{\mathrm{all}}$ reflects the estimate of $\beta_{\mathrm{AGE}}$ , with the SE calculated over all 2,000 simulation replicates; ${\hat{\beta }}_{\mathrm{naive}}$ , ${\hat{\beta }}_{\mathrm{boot}}$ , and ${\hat{\beta }}_{\mathrm{like}}$ reflect the averages (SEs) over only significant replicates (where CMC Wald-test p<0.05 ) for the naive method of taking the same average of the $\beta_{\mathrm{AGE}}$ directly and the bootstrap- and likelihood-based bias correction methods, respectively, as described in Section 2.3. The underlying individual effect $\beta_{j}$ (MAFs $q_{j}$ ) = [0(0.0016), 0.97(0.0047), 0.83(0.0031), 0.97(0.0016), 0.83(0.0016), 0(0.0016), 0(0.0016), 0.93(0.0016), 0.53(0.0031), 0.93(0.0016), 0.53(0.0047)], where, under the bidirectional scenario, the signs for $\beta_{8}$ and $\beta_{10}$ are flipped.

Description	Eight causal in the same direction	Eight causal with 2° in the opposite direction
$\beta_{\text{AGE}}$	0.64	0.42
Estimation, mean (SE), before significance testing across all 2,000 replicates		
${\hat{\beta }}_{\text{all}}$	0.72 (0.27)	0.48 (0.27)
Estimation, mean (SE), after significance testing across 528 or 254 replicates		
	Power = 1,527/2000 = 76.3%	Power = 844/2000 = 42.2%
${\hat{\beta }}_{\text{naive}}$	0.83 (0.20)	0.72 (0.15)
${\hat{\beta }}_{\text{boot}}$	0.66 (0.33)	0.46 (0.29)
${\hat{\beta }}_{\text{like}}$	0.65 (0.34)	0.46 (0.30)

First, without conditioning on the association testing results, the parameter estimation is unbiased, as expected: ${\hat{\beta }}_{\mathrm{all}}$ is 0.34, close to 0.3, the true $\beta_{\mathrm{AGE}}$ value for the unidirectional scenario, and it is 0.22 compared with 0.19 for the bidirectional scenario.

Second, when the average estimate was calculated among only the significant replicates, bias in ${\hat{\beta }}_{\mathrm{all}}$ was considerable due to the winner’s curse: ${\hat{\beta }}_{\mathrm{naive}}-\beta_{\mathrm{AGE}}=0.67-0.3=0.37$ and =0.64−0.19=0.45 for the unidirectional and bidirectional scenarios, respectively, and as expected, the bias increases as power decreases.

**FIGURE 1:**
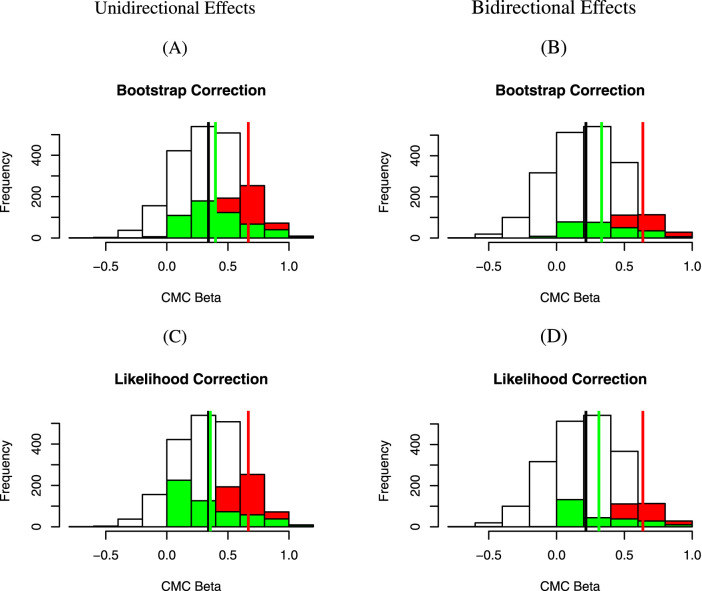
Distributions of $\beta_{\mathrm{AGE}}$ estimates before and after the corrections. **(A–C)** Settings where all causal variants have the same direction of effects (unidirectional). **(B–D)** Bidirectional effects. The underlying individual effect $\beta_{j}$ (MAFs $q_{j}$ ) = [0(0.0016), 0(0.0047), 0.83(0.0031), 0.97(0.0016), 0(0.0016), 0(0.0016), 0(0.0016), 0(0.0016), 0(0.0031), 0.93(0.0016), 0.53(0.0047)], where, under the bidirectional scenario, the sign for $\beta_{10}$ is flipped. The vertical black line represents the empirical estimate of the “true” AGE value, ${\hat{\beta }}_{\mathrm{all}}$ , the average of the effect size estimates from all 2,000 simulated replicates (white bars); ${\hat{\beta }}_{\mathrm{naive}}$ (vertical red line) reflects the average of the effect size estimates from the significant replicates with CMC rare variant testing Wald p<0.05 ; ${\hat{\beta }}_{\mathrm{corrected}}$ (vertical green line) reflects the average of the effect size estimates following correction using the bootstrap method **(A,B)** or the approximate likelihood method **(C,D)**. See Table 1 for a numerical summary.

Third, similar to the results of Liu and Leal (2012), the bootstrap bias adjustment works well under the CMC RV association testing method. Using the bootstrap correction (Liu and Leal, 2012), the average absolute bias owing to the winner’s curse was greatly reduced, ${\hat{\beta }}_{\mathrm{boot}}-\beta_{\mathrm{AGE}}=0.40-0.30=0.10$ and =0.33−0.19=0.14 for the unidirectional and bidirectional scenarios, respectively (Table 1; Figures 1A,B). Of note, the results of using the mean of the bootstrap samples [as originally proposed by Sun and Bull (2005)], instead of the median [as used by Liu and Leal (2012)], were not noticeably different from each other. Thus, only results using median bootstrap bias estimates are reported in this study.

Fourth, the approximate likelihood approach proposed by Ghosh et al. (2008) appears to perform as well as, if not better than, the bootstrap approach, where ${\hat{\beta }}_{\mathrm{like}}-\beta_{\mathrm{AGE}}=0.36-0.30=0.06$ and =0.31−0.19=0.12 (Table 1; Figures 1C,D). We observed one exception to this; under the scenario that included very large effects in opposing directions (Table 2), the likelihood method demonstrated greater bias $\left({\hat{\beta }}_{\mathrm{like}}-\beta_{\mathrm{AGE}}=0.27-0.13=0.14\right)$ than the bootstrap approach $\left({\hat{\beta }}_{\mathrm{boot}}-\beta_{\mathrm{AGE}}=0.20-0.13=0.07\right)$ . Due to its ease of implementation and computational efficiency, the likelihood approach may be preferred over the bootstrapping approach.

Finally, it is important to point out that $\beta_{\mathrm{AGE}}$ does not reflect the true average effect size of the causal variants $\beta_{\mathrm{AGE,causal}}$ . $\beta_{\mathrm{AGE}}$ is attenuated compared with $\beta_{\mathrm{AGE,causal}}$ due to the inclusion of variants with no effects (i.e., $\beta_{j}=0$ ). For example, in the unidirectional effect scenario, while $\beta_{\mathrm{AGE}}=0.3$ [the MAF-weighted average of effect across all 11 RVs, based on Equation 2], $\beta_{\mathrm{AGE,causal}}=0.74$ when considering only the four causal RVs. Additionally, the effect direction also affects the interpretation of $\beta_{\mathrm{AGE,causal}}$ . For example, under the bidirectional effect scenario where the effect sizes are $\beta_{3}=0.83$ , $\beta_{4}=0.97$ , $\beta_{10}=-0.93$ , and $\beta_{11}=0.53$ , the average effect size among the four causal effects is $\beta_{\mathrm{AGE,causal}}=0.47$ , which is substantially smaller than the average *magnitude* of the four individual effects. This is not surprising considering the definition of $\beta_{\mathrm{AGE}}$ , which is sensitive to the direction of the effect.

### 3.3 Bias–variance tradeoff in effect estimation

Estimates of effect sizes, such as the bias-corrected estimates investigated in this study, require standard error estimates for inference procedures. Ghosh et al. (2008) constructed confidence intervals with correct conditional coverage by applying the original Neymanian concept of confidence regions, where intervals are derived from the known conditional distribution of the test statistic after selection. Specifically, the acceptance region A(μ,1−α) is defined between α/2 and 1−α/2 quantiles of the conditional density $p_{\mu }\left(z||Z|>c\right)$ , ensuring exact coverage probability 1−α for any μ .

Although Liu and Leal (2012) did not propose methods for calculating confidence intervals (CIs) or standard errors (SEs) for the bootstrap-corrected estimates of $\beta_{\mathrm{AGE}}$ , the two-level bootstrap sampling scheme described by Faye et al. (2011) for common GWAS variants could be adapted to rare variant analyses to provide resampling-based standard error estimators for $\beta_{\mathrm{AGE}}$ , thus providing a basis for inference.

Rather than investigating and reporting such standard error estimation methods, we report the empirical standard error estimates to investigate the bias–variance trade-off related to the bias-corrected estimate of $\beta_{\mathrm{AGE}}$ . We find that bias-correction methods result in increased standard errors compared to the uncorrected “naive” estimates across all scenarios (Tables 1‐3). Interestingly, the SE estimates for the bias-corrected estimates closely approximate those of the unbiased estimates ${\hat{\beta }}_{\mathrm{all}}$ , which represent the true underlying effect sizes and standard deviations.

### 3.4 Individual variant effects

A limitation of the current state of the rare variant literature is a demonstration of the bias associated with the *individual* causal effects contributing to the winner’s curse bias. To this end, we examined the individual effect estimates of the causal variants over the GAW17 simulation replicates to shed light on some of the contributing factors that might play a role in the different scenarios of pooled effects. To this end, we first applied three RV association methods: CMC (Li and Leal, 2008), CAST (Morgenthaler and Thilly, 2007), and C-Alpha (Neale et al., 2011), jointly testing a group of variants. We then summarized the individual variant effect estimates, with or without conditioning on the testing results.

Results of the individual effect estimates ( ${\hat{\beta }}_{j}$ , j=3,4,10,11 ; the four causal variants) are summarized in Table 4 for RV joint testing based on CMC, CAST, and C-Alpha. The average ${\hat{\beta }}_{j}$ over all replicates and the average over only the significant (the association *p*-values < 0.05) replicates are presented for each of the four causal SNPs under each of the two scenarios (unidirectional and bidirectional effects). As both the pooled variant Wald (CMC) and linear combined score (CAST) statistics represent the linear class of RV joint testing approach, the results of the individual variant effect analyses conditional on these two testing methods are quite similar and thus discussed jointly below. This similarity in the linear class methods’ results suggests that we could expect comparable outcomes among quadratic class methods, such as those observed with C-Alpha.

**TABLE 4:** Individual causal variant β estimate summary conditional on significant linear (CMC and CAST) or quadratic (C-Alpha) association testing. The underlying individual effect $\beta_{j}$ (MAFs $q_{j}$ ) = [0(0.0016), 0(0.0047), 0.83(0.0031), 0.97(0.0016), 0(0.0016), 0(0.0016), 0(0.0016), 0(0.0016), 0(0.0031), 0.93(0.0016), 0.53(0.0047)], where, under the bidirectional scenario, the sign for $\beta_{10}$ is flipped. ${\hat{\beta }}_{\mathrm{all}}$ is the average $\beta_{j}$ estimates over all 2,000 replicates, and ${\hat{\beta }}_{\mathrm{naive}}$ is averaged over only the significant replicates (p<0.05) . Bias is the effect estimation bias of ${\hat{\beta }}_{\mathrm{naive}}$ , where the positive bias represents *upward* bias away from 0, while the *negative bias* represents *downward* bias toward 0. The relative bias (rel.bias) is the bias divided by true $\beta_{j}$ .

Association testing based on CMC (Liu and Leal, 2012)									
		Four causal in the same direction				Four causal with 1° in the opposite direction			
		Power = 528/2000 = 26.4%				Power = 254/2000 = 12.7%			
RV index	True $\beta_{j}$	${\hat{\beta }}_{\mathrm{all}}$	${\hat{\beta }}_{\mathrm{naive}}$	bias	rel.bias	${\hat{\beta }}_{\mathrm{all}}$	${\hat{\beta }}_{\mathrm{naive}}$	bias	rel.bias
3	0.83	0.80	1.06	0.23	0.28	0.81	1.22	0.39	0.47
4	0.97	0.98	1.37	0.40	0.41	0.99	1.34	0.37	0.38
10	0.93 or −0.93	0.93	1.24	0.31	0.33	−0.94	−0.51	−0.42	−0.45
11	0.53	0.50	0.82	0.29	0.55	0.51	0.93	0.40	0.75

Association testing based on CAST (Morgenthaler and Thilly, 2007)									
		Four causal in the same direction				Four causal with 1° in the opposite direction			
		Power = 536/2000 = 26.8%				Power = 302/2000 = 15.1%			
RV index #	True $\beta_{j}$	${\hat{\beta }}_{\mathrm{all}}$	${\hat{\beta }}_{\mathrm{naive}}$	bias	rel.bias	${\hat{\beta }}_{\mathrm{all}}$	${\hat{\beta }}_{\mathrm{naive}}$	bias	rel.bias
3	0.83	0.80	1.04	0.21	0.25	0.81	1.12	0.29	0.35
4	0.97	0.98	1.55	0.58	0.60	0.99	1.61	0.64	0.66
10	0.93 or −0.93	0.93	1.18	0.25	0.27	−0.94	−0.56	−0.37	−0.40
11	0.53	0.50	0.78	0.25	0.47	0.51	0.83	0.30	0.57

Association testing based on C-Alpha (Neale et al., 2011)									
		Four causal in the same direction				Four causal with 1° in the opposite direction			
		Power = 400/2000 = 20%				Power = 410/2000 = 20.5%			
RV index #	$\beta_{j}$	${\hat{\beta }}_{\mathrm{all}}$	${\hat{\beta }}_{\mathrm{naive}}$	bias	rel.bias	${\hat{\beta }}_{\mathrm{all}}$	${\hat{\beta }}_{\mathrm{naive}}$	bias	rel.bias
3	0.83	0.80	1.17	0.34	0.41	0.81	1.16	0.33	0.40
4	0.97	0.98	1.49	0.52	0.54	0.99	1.50	0.53	0.55
10	0.93 or −0.93	0.93	1.05	0.12	0.13	−0.94	−1.18	0.25	0.27
11	0.53	0.50	0.89	0.36	0.68	0.51	0.90	0.37	0.70

Consistent with the previous literature on the power of RV association testing, when all causal SNPs have the same direction of the effect, the linear test has a slight advantage, but the power of the quadratic test is comparable (power of 20% for C-Alpha compared with 0.27 and 0.26 for CAST and CMC, respectively). On the other hand, when one of the four causal SNPs (25%) has an effect in the opposite direction to the others, the power is greater with the quadratic test statistic (0.21 for C-Alpha compared with 0.15 and 0.13 for CAST and CMC, respectively). Moreover, consistent with the previous literature on the winner’s curse for common variants, the relative bias increases as power decreases.

However, under the linear testing approaches (CMC and CAST), causal variants with effects in opposite directions (right side of Table 4) led to an increased upward bias for the individual ${\hat{\beta }}_{\mathrm{naive}}$ in the majority group (3 of 4 SNPs sharing the same direction of effect) and a large *downward bias (shrinkage toward 0)* in the magnitude of the minority group’s ${\hat{\beta }}_{\mathrm{naive}}$ (1 of 4 SNPs with the opposite direction of the effect), which is previously unreported. Intuitively, causal variants with an effect in the opposite direction (the minority group) dilute the collapsed genotype effect (direction of the majority group) on average for a given linear testing procedure. Thus, when the observed effects of these oppositional variants are smaller, the collapsed genotype effect driven by the majority group is more likely to be significant. Additionally, due to these oppositional effects, the majority group’s causal variants need to demonstrate an even stronger observed effect size for the linear testing procedure to yield a significant pooled genotype effect.

Unlike linear testing methods, quadratic testing (C-Alpha) involving causal variants with effects in opposite directions does not significantly alter the magnitudes of biases for the individually estimated $\beta_{j}$ compared to the scenario where all causal SNP effects are unidirectional. Under the quadratic testing, the winner’s curse bias yields an increased magnitude of bias for all individual SNP effect estimates. This is also intuitive as the squaring method inherent in the quadratic test ensures that each of the causal variants will contribute to a significant test, regardless of which direction the extreme/large effects point to. This is also reflected by the unchanged power from the unidirectional scenario to the bidirectional scenario (both approximately 20%), unlike the linear testing methods.

## 4 Discussion

The upward bias in effect size estimation due to selective inference conditional on significant association testing is well understood for common variants and has been demonstrated for rare variants in the context of $\beta_{\mathrm{AGE}}$ , the average effect across all RVs analyzed. However, the possibility of downward estimation bias due to heterogeneous effect directions among the RVs and its relationship with the RV association testing methods used has previously gone unrecognized. We investigated the combination of these factors through a simulation study of rare variant testing and estimation based on GAW17 data.

Starting with previously defined $\beta_{\mathrm{AGE}}$ , we first demonstrated the upward bias in ${\hat{\beta }}_{\mathrm{naive}}$ , the naive estimate, replicating the earlier study of Liu and Leal (2012)). We then demonstrated that the conditional likelihood method and the original bootstrap method proposed for common variants work well in providing bias-corrected estimates, in addition to the modified bootstrap method of Liu and Leal (2012)).

We then examined how heterogeneous effect direction affects our interpretation of $\beta_{\mathrm{AGE}}$ , along with the estimation of $\beta_{j}$ , the *individual* variant effect size.

First, as $\beta_{\mathrm{AGE}}$ is an MAF-weighted *linear* average of $\beta_{j}$ , opposite effect directions result in a pessimistic $\beta_{\mathrm{AGE}}$ . To check this, consider the simplistic case of two causal variants with identical MAF and effect magnitude but in opposite effect directions; then, $\beta_{\mathrm{AGE}}=0$ . Thus, the choice of how to define the average genetic effect across a set of variants is important. One useful direction for future research is the examination of the behavior of the non-centrality parameter of the association test statistic, which not only depends on both MAF and $\beta_{j}$ but is also directly related to power, which, in turn, is inversely associated with estimation bias.

Second, our simulation results showed that individual SNP effect estimate bias depends not only on the directionality of SNPs within the set but also on the choice of linear or quadratic association testing approach. For linear tests, individual estimates ( ${\hat{\beta }}_{j}$ s) can exhibit both upward and *downward* biases, depending on the sign of the underlying effects ( $\beta_{j}$ s). For quadratic tests, the individual estimates are always upwardly biased (i.e., always larger in magnitude).

Although our simulation studies only focused on CAST (Morgenthaler and Thilly, 2007) from the linear class and C-Alpha (Neale et al., 2011) from the quadratic class, the general analytical form of these tests suggests that our observations can be generalized to other specific tests from each class, e.g., WSS (Madsen and Browning, 2009) from the linear class and SKAT (Wu et al., 2011) from the quadratic class. The behaviors of tests from the hybrid class, such as SKAT-O (Lee et al., 2012) and Fisher’s method (Derkach et al., 2013), however, are unknown and warrant future studies.

As rare variant studies scale to exome- and genome-wide datasets, computational efficiency becomes increasingly important. Bootstrap-based methods, while flexible, can be computationally expensive for large-scale analyses. Likelihood-based corrections, as evaluated in our study, offer a more scalable alternative, particularly when computational resources are limited. Additionally, for extremely rare variants (e.g., minor allele frequency <0.1% ), a key challenge in bootstrap resampling ensures that both subsamples contain at least one instance of the effect allele. Random splits may lead to scenarios where the effect allele is absent in one subsample, potentially distorting the bootstrap distribution. A possible mitigation strategy is stratified resampling, where subsamples are selected to ensure the representation of effect alleles. However, intentional selection of subsamples risks introducing bias and is beyond the scope of this work.

Existing bias-correction methods for common variants can be effectively applied to estimate the AGE for rare variants. However, pooling rare variants presents challenges for estimating individual effect sizes, particularly when causal variants have bidirectional effects. As rare variant studies expand with whole-genome sequencing and biobank-scale datasets, future work should focus on improving bias-correction methods for both pooled and individual effect estimates. Although likelihood-based methods are efficient, machine learning and Bayesian approaches could provide more flexible models for capturing complex effect architectures. Additionally, incorporating corrected rare variant effects into polygenic risk scores may enhance predictive accuracy, especially for diseases with strong rare variant contributions. Developing methods that optimally combine linear and variance-component tests, such as SKAT-O, could further improve estimation in the presence of effect heterogeneity. Advancing these techniques will strengthen our ability to quantify rare variant contributions to complex traits and improve genetic risk prediction.

## Data Availability

The “simulated” datasets presented in this study can be found at: https://github.com/dsoave/WinnersCurse_RareVariants_Public.
